# Supplementary figures and images for: Identification of a non-canonical ciliate nuclear genetic code where UAA and UAG code for different amino acids
Source: PLoS Genet. 2023 Oct 5;19(10):e1010913. doi: 10.1371/journal.pgen.1010913 (PMC10553269; doi:10.1371/journal.pgen.1010913)

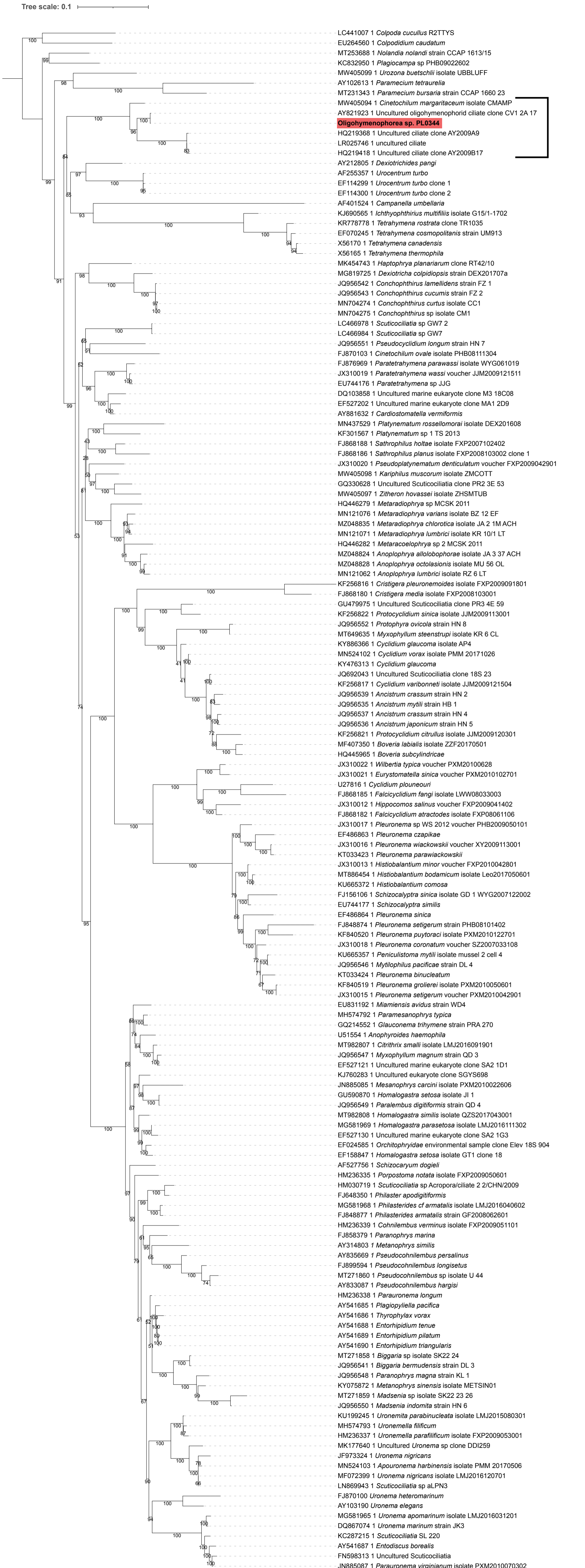

Supplement: S1 Fig — (PDF) [file pgen.1010913.s001.pdf]

# All Genes

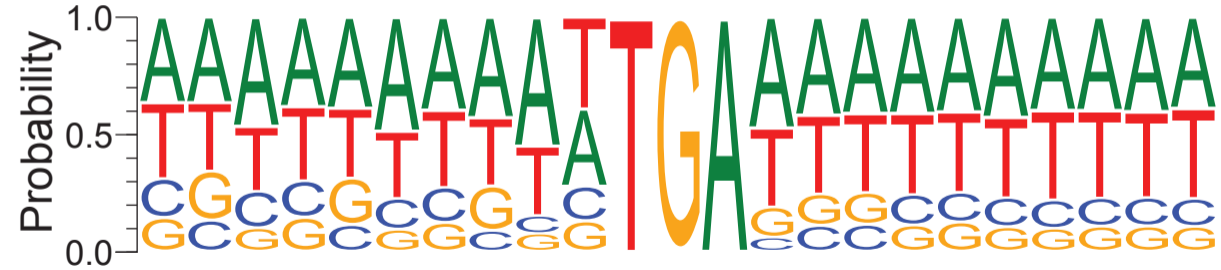

# Highly Expressed Genes

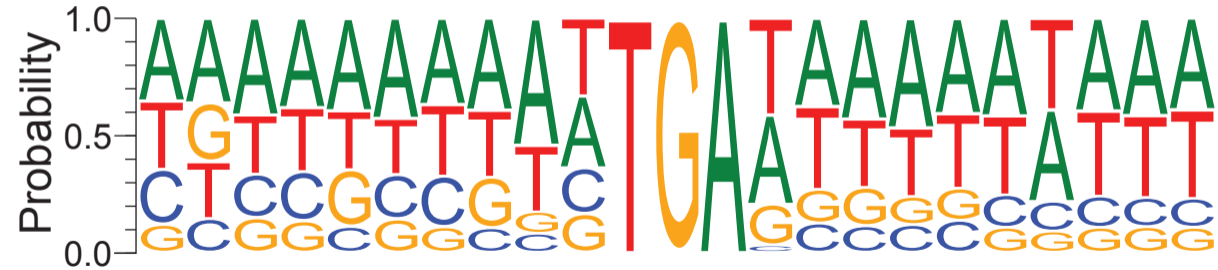

Supplement: S3 Fig — (PDF) [file pgen.1010913.s003.pdf]

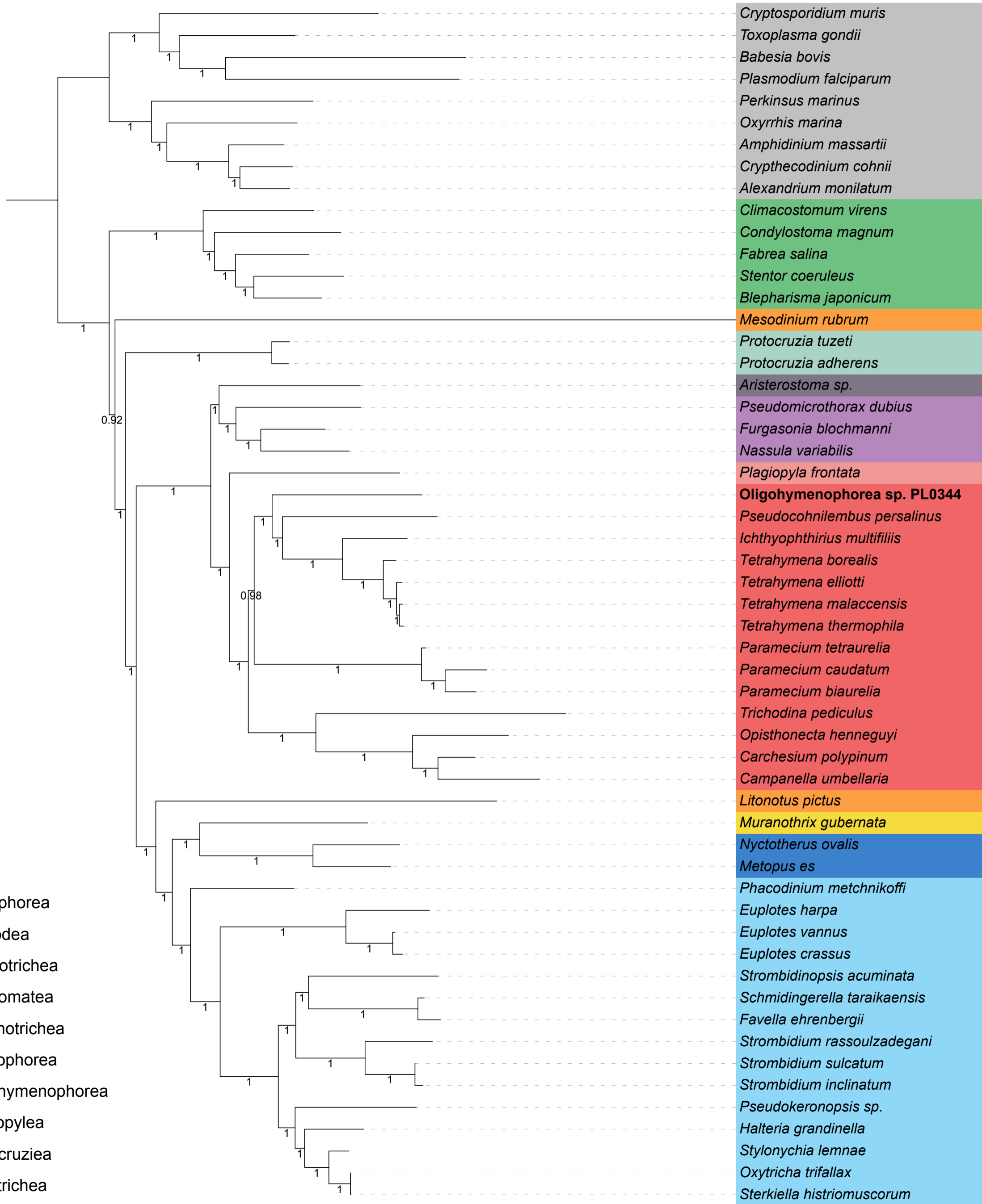

Supplement: S4 Fig — (PDF) [file pgen.1010913.s004.pdf]

A) TM9SF1

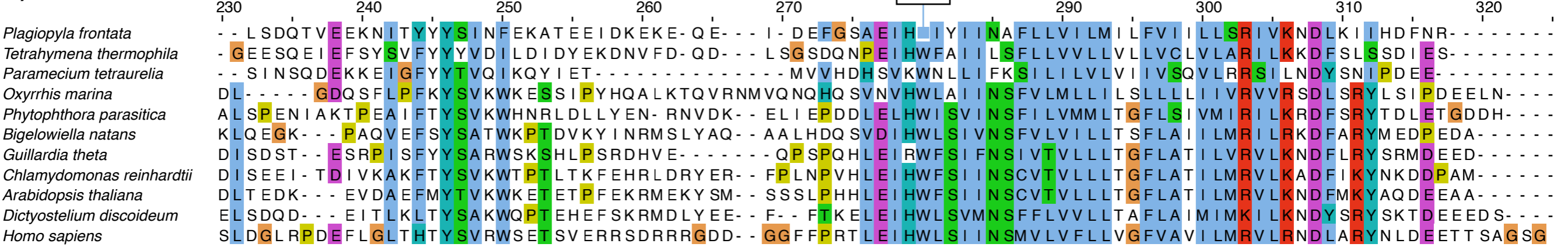

B) PIK3C3

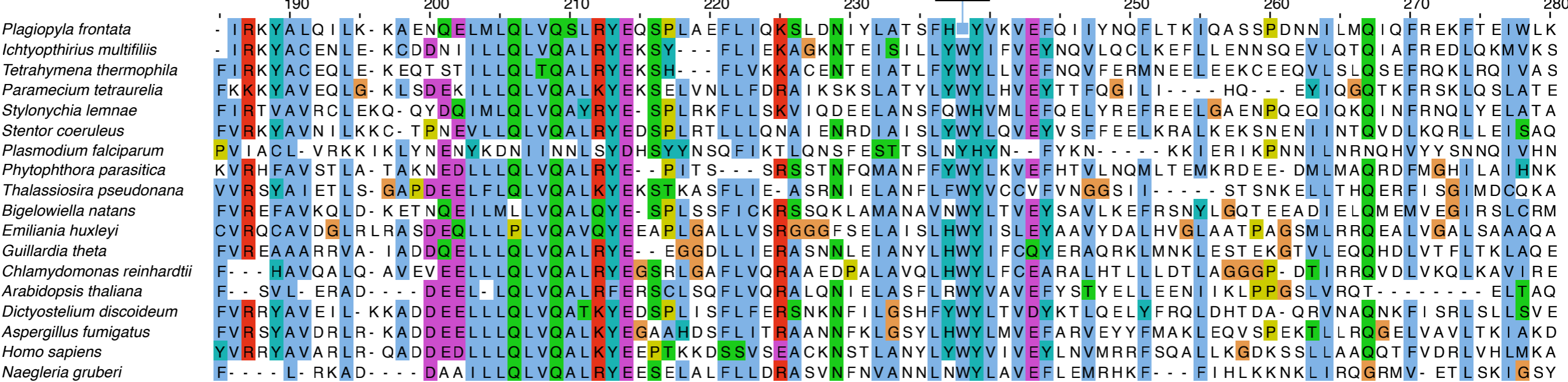

C) CRNL1

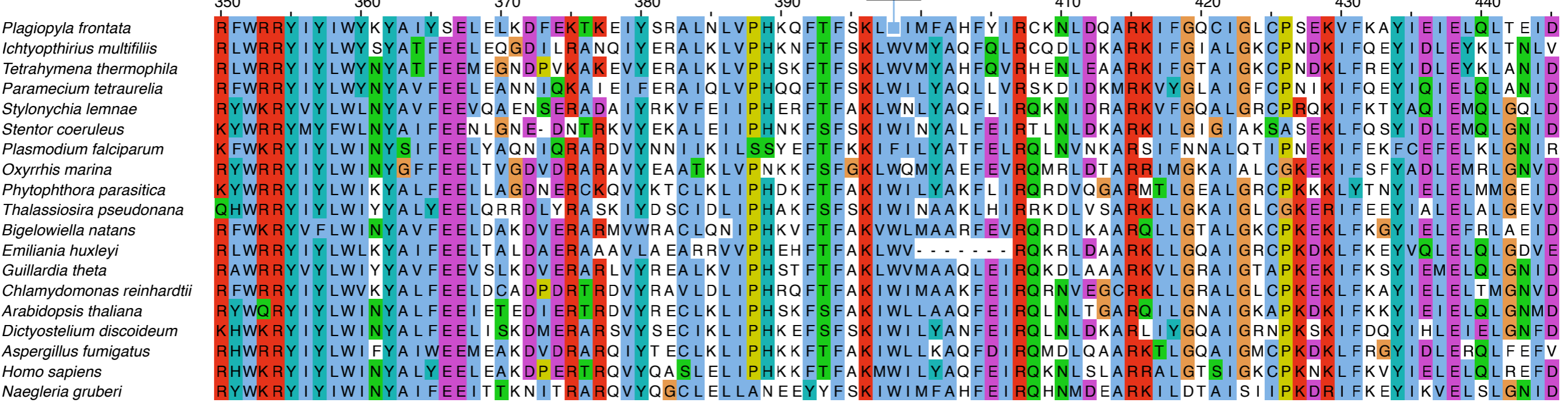

Supplement: S5 Fig — A) TM9SF1. B) PIK3C3. C) CRNL1. (PDF) [file pgen.1010913.s005.pdf]
