# Supplementary material for: Identification of a non-canonical ciliate nuclear genetic code where UAA and UAG code for different amino acids
Source: PLoS Genet. 2023 Oct 5;19(10):e1010913. doi: 10.1371/journal.pgen.1010913 (PMC10553269; doi:10.1371/journal.pgen.1010913)
Supplement: S2 Fig — tRNA genes predicted to be pseudogenes, contain introns, or that were excessively truncated were excluded. Lysine, glutamic acid, and suppressor tRNA genes are highlighted. (PDF) [file pgen.1010913.s002.pdf]

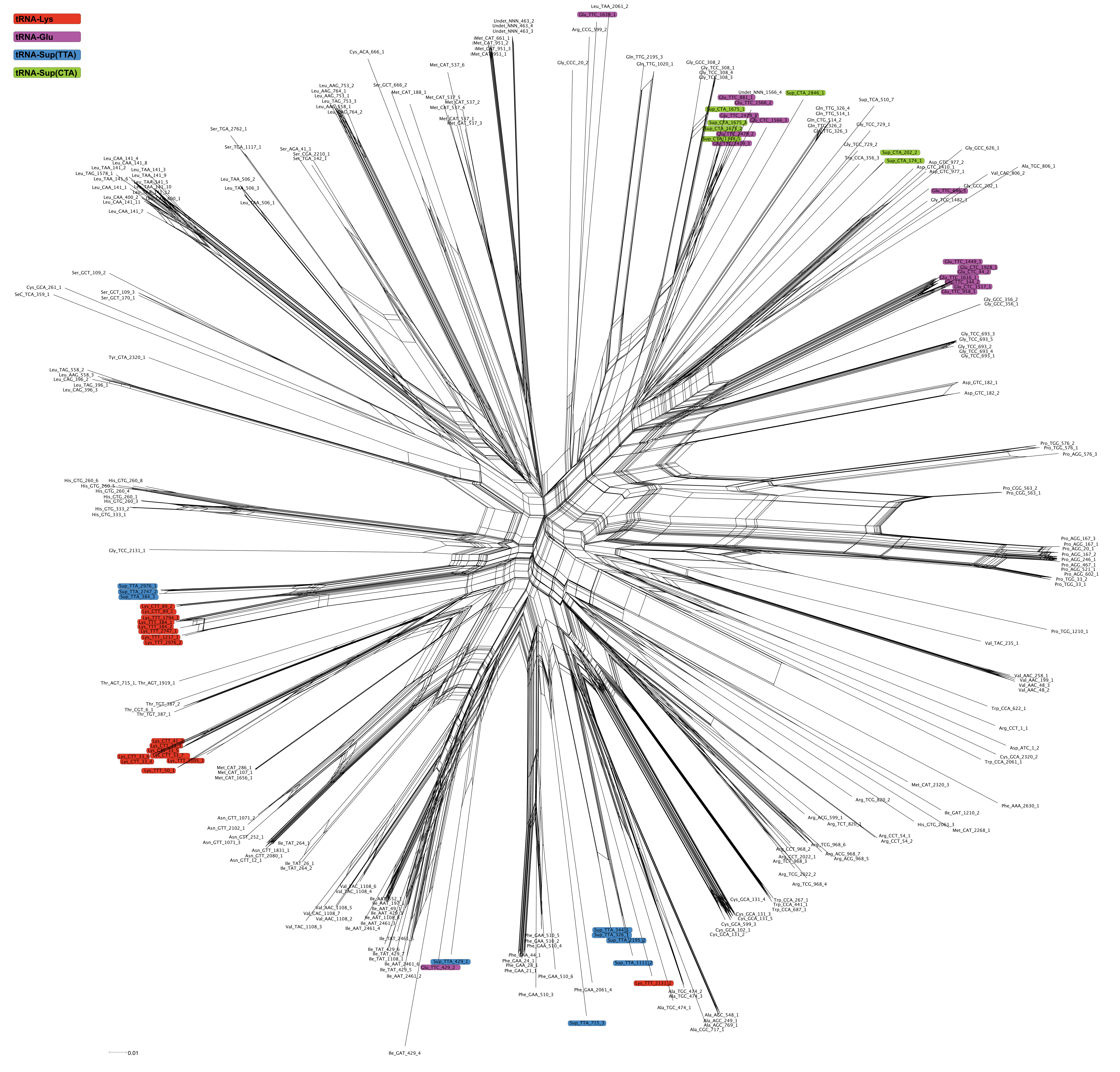

tRNA-Lys
tRNA-Glu
tRNA-Sup(TTA)
tRNA-Sup(CTA)
Leu_TAA_2061_2
Glu_TTC_1639_1
Arg_CCG_599_2
Undet_NNN_463_2
Undet_NNN_463_4
Undet_NNN_463_3
iMet_CAT_661_1
iMet_CAT_951_2
iMet_CAT_951_3
Cys_ACA_666_1
Met_CAT_537_6
Gly_CCC_20_2
Gln_TTG_2195_3
Gln_TTG_1020_1
Gly_GCC_308_2
Gly_TCC_308_1
Gly_TCC_308_4
Gly_TCC_308_3
Undet_NNN_1566_4
Sup_CTA_2846_1
Glu_TTC_881_1
Glu_TTC_1566_2
Sup_CTA_1675_1
Glu_TTC_2479_1
Glu_CTC_1566_3
Sup_CTA_1675_3
Sup_CTA_1675_2
Glu_TTC_2479_2
Glu_TTC_2479_3
Sup_TCA_510_7
Gln_TTG_326_4
Gln_TTG_514_1
Gln_CTG_514_2
Gln_TTG_326_2
Gln_TTG_326_3
Gly_TCC_729_1
Gly_TCC_729_2
Trp_CCA_356_3
Sup_CTA_202_2
Sup_CTA_174_1
Gly_GCC_626_1
Asp_GTC_977_2
Asp_GTC_1410_1
Asp_GTC_977_1
Ala_TGC_806_1
Val_CAC_806_2
Gly_GCC_202_1
Glu_TTC_646_1
Gly_TCC_1482_1
Leu_AAG_753_2
Leu_AAG_764_1
Leu_AAG_753_1
Leu_TAG_753_3
Leu_AAG_558_1
Leu_CAG_764_2
Ser_GCT_666_2
Met_CAT_188_1
Met_CAT_537_5
Met_CAT_537_2
Met_CAT_537_4
Met_CAT_537_1
Met_CAT_537_3
Ser_TGA_2762_1
Ser_TGA_1117_1
Ser_AGA_41_1
Ser_CGA_2210_1
Ser_TGA_142_1
Leu_CAA_141_4
Leu_CAA_141_8
Leu_TAA_141_2
Leu_TAA_141_3
Leu_TAG_1578_1
Leu_TAA_141_9
Leu_TAA_141_6
Leu_TAA_141_5
Leu_CAA_141_1
Leu_TAA_141_10
Leu_CAA_400_2
Leu_CAA_141_11
Leu_CAA_141_7
Leu_TAA_506_2
Leu_TAA_506_3
Leu_TAA_506_1
Glu_TTC_1449_1
Glu_CTC_1928_1
Glu_CTC_44_2
Glu_TTC_1616_1
Glu_CTC_344_2
Glu_CTC_1517_1
Glu_TTC_958_1
Gly_GCC_356_2
Gly_GCC_356_1
Ser_GCT_109_2
Cys_GCA_261_1
SeC_TCA_359_1
Ser_GCT_109_3
Ser_GCT_170_1
Gly_TCC_693_3
Gly_TCC_693_5
Gly_TCC_693_2
Gly_TCC_693_4
Gly_TCC_693_1
Tyr_GTA_2320_1
Leu_TAG_558_2
Leu_AAG_558_3
Leu_CAG_396_2
Leu_TAG_396_1
Leu_CAG_396_3
Asp_GTC_182_1
Asp_GTC_182_2
Pro_TGG_576_2
Pro_TGG_576_1
Pro_AGG_576_3
His_GTG_260_6
His_GTG_260_8
His_GTG_260_5
His_GTG_260_4
His_GTG_260_1
His_GTG_260_3
His_GTG_333_2
His_GTG_333_1
Pro_CGG_563_2
Pro_CGG_563_1
Gly_TCC_2131_1
Pro_AGG_167_3
Pro_AGG_167_1
Pro_AGG_20_1
Pro_AGG_167_2
Pro_AGG_246_1
Pro_AGG_467_1
Pro_AGG_521_1
Pro_AGG_602_1
Pro_TGG_33_2
Pro_TGG_33_1
Sup_TTA_2976_1
Sup_TTA_2747_2
Sup_TTA_384_3
Lys_CTT_89_2
Lys_CTT_89_1
Lys_TTT_1794_1
Lys_TTT_384_1
Lys_TTT_384_2
Lys_TTT_2747_1
Lys_TTT_1217_1
Lys_TTT_2976_2
Pro_TGG_1210_1
Val_TAC_235_1
Val_AAC_258_1
Val_AAC_199_1
Val_AAC_48_3
Val_AAC_48_2
Thr_AGT_715_1, Thr_AGT_1919_1
Thr_TGT_387_2
Thr_CGT_6_1
Thr_TGT_387_1
Trp_CCA_622_1
Arg_CCT_1_1
Lys_CTT_41_2
Lys_CTT_33_1
Lys_CTT_33_6
Lys_CTT_33_4
Lys_CTT_33_7
Lys_TTT_1105_1
Lys_TTT_50_1
Asp_ATC_1_2
Cys_GCA_2320_2
Trp_CCA_2061_1
Met_CAT_286_1
Met_CAT_107_1
Met_CAT_1656_1
Met_CAT_2320_3
Phe_AAA_2630_1
Arg_TCG_820_2
Ile_GAT_1210_2
His_GTG_2061_3
Met_CAT_2268_1
Arg_ACG_599_1
Arg_TCT_820_1
Arg_CCT_54_1
Arg_CCT_54_2
Asn_GTT_1071_2
Asn_GTT_2102_1
Asn_GTT_252_1
Asn_GTT_1071_3
Ile_TAT_264_1
Asn_GTT_1831_1
Asn_GTT_2080_1
Asn_GTT_12_1
Ile_TAT_26_1
Ile_TAT_264_2
Arg_TCG_968_6
Arg_CCT_968_2
Arg_CCT_2022_1
Arg_TCT_968_3
Arg_ACG_968_7
Arg_ACG_968_5
Arg_TCG_2022_2
Arg_TCG_968_4
Val_TAC_1108_6
Val_TAC_1108_4
Val_AAC_1108_5
Val_CAC_1108_7
Val_AAC_1108_2
Val_TAC_1108_3
Ile_AAT_552_1
Ile_AAT_197_1
Ile_AAT_49_1
Ile_AAT_429_3
Ile_AAT_1108_8
Ile_AAT_2461_3
Ile_AAT_2461_4
Ile_TAT_2461_5
Ile_TAT_429_6
Ile_TAT_429_7
Ile_TAT_1108_1
Ile_AAT_2461_6
Ile_TAT_429_5
Ile_AAT_2461_2
Sup_TTA_429_1
Glu_TTC_429_2
Ile_GAT_429_4
Cys_GCA_131_4
Cys_GCA_131_3
Cys_GCA_131_5
Cys_GCA_599_3
Cys_GCA_102_1
Cys_GCA_131_2
Trp_CCA_267_1
Trp_CCA_441_1
Trp_CCA_687_1
Phe_GAA_510_5
Phe_GAA_510_2
Phe_GAA_510_4
Phe_GAA_44_1
Phe_GAA_24_1
Phe_GAA_28_1
Phe_GAA_21_1
Phe_GAA_510_6
Phe_GAA_510_3
Phe_GAA_2061_4
Sup_TTA_344_1
Sup_TTA_326_1
Sup_TTA_2195_2
Sup_TTA_1111_2
Lys_TTT_2131_2
Ala_TGC_474_2
Ala_TGC_474_3
Ala_TGC_474_1
Ala_AGC_548_1
Ala_AGC_249_1
Ala_AGC_769_1
Ala_CGC_717_1
Sup_TTA_715_3
0.01
