# Supplementary material for: Identification of a non-canonical ciliate nuclear genetic code where UAA and UAG code for different amino acids
Source: PLoS Genet. 2023 Oct 5;19(10):e1010913. doi: 10.1371/journal.pgen.1010913 (PMC10553269; doi:10.1371/journal.pgen.1010913)
Supplement: Attachment — Submitted filename: Response Letter - Review Commons full-revision.docx [file pgen.1010913.s009.docx]

**Manuscript number:** RC-2023-01855

**Corresponding author(s):**

Dr David Swarbreck,

Professor Neil Hall,

Professor Thomas Richards

## 1. General Statements [optional]

*This section is optional. Insert here any general statements you wish to make about the goal of the study or about the reviews.*

**We would like to thank the three reviewers for their thorough review of our manuscript and their detailed comments and very helpful suggestions to improve the manuscript. Overall, we thought the reviews were very positive with the reviewers commenting that our discovery of a novel genetic code variant is a “cause for celebration” and that our study is “technically solid” and “rigorous”. All three reviewers agree that our manuscript would “stimulate new discussions in the field of genetic code evolution” and also be of broad interest to evolutionary cell biologists, protistologists and the translation/protein synthesis community at large. The reviewers highlight the particular novelty of the genetic code variant described here due to it being an exception to the wobble hypothesis which adds a new level of complexity to stop-codon reassignment. The reviewers share our frustration about the lack of proteomics data due to being unable to establish a stable culture but acknowledge that we address this limitation frankly in our discussion and agree that it is “frustrating but it's not a limitation”.**

**We present an updated and improved version of the manuscript after taking on board the reviewers’ suggestions. Our point-by-point responses to their comments and our modifications are detailed below in bold.**

## 2. Point-by-point description of the revisions

*This section is mandatory.* *Please insert a point-by-point reply describing the revisions that were already carried out and included in the transferred manuscript.*

**Reviewer #1 (Evidence, reproducibility and clarity (Required)):**

Summary

This study by J. McGowan and colleagues reports the discovery of a ciliate species that uses a variant genetic code where the codons UAA and UAG, which are stop codons in the canonical code, instead code for lysine and glutamate respectively. The primary data are genomic and transcriptomic sequence libraries from single cells. The genetic code was predicted by aligning coding sequences to references from other species and examining the most frequent amino acids in positions homologous to putative coding-UAA/UAGs. They also identified suppressor tRNAs for UAA and UAG, and tandem in-frame stop UGAs (but not UAA/UAG) in the 3'-UTR, which further support the recoding of UAA and UAG.

A limitation of this study (and several other recent studies on variant genetic codes) is that the predictions are based on nucleic acid sequencing, without confirmation from proteomics. The authors acknowledge and briefly but frankly discuss the limitations in their manuscript (lines 258-261).

Major comments

Controls against contamination and sequence chimeras

The ciliate species studied here was an environmental isolate, and sequence libraries were prepared by amplification from small pools of cells sorted by FACS. The genome assembly was produced by co-assembly of multiple amplified libraries. Given the potential for contamination and amplification artefacts (such as sequence chimeras) associated with these methods, I think it is important to demonstrate that the data truly originate from one species, so as to rule out the possibility that the co-assembly may be chimeric, i.e. representing two or more organisms with different genetic codes (one with UAA recoded and the other with UAG recoded, for instance). Even if the cell sorting was accurate, contamination could still enter down the line during library preparation so it would be important to show internal evidence from the sequence data too.

**We understand the reviewer's concerns about the possibility of contamination as it can be a major issue in environmental single cell sequencing experiments. We have addressed the individual points below in detail to demonstrate that we have generated a clean genome assembly of a single ciliate species but also summarise here:**

- **The cells we sequenced originated from the same clonally isolated cell propagated in culture**
- **We have manually curated the assembly**
- **The assembly has a unimodal GC content peak with a low BUSCO duplication score**
- **Most genes (95.9 %) contain both in-frame UAA and UAG codons**
- **We recovered a single identical ciliate 18S rRNA gene across all 10 samples**
- **De novo assemblies of the 10 individual gDNA libraries are virtually identical in terms of average nucleotide identity**
- **We also predicted the genetic code for each of the genome and transcriptome samples individually**
- **>85% of the final assembly is taxonomically classified as Ciliophora. The remainder is either unclassified (i.e. no hits) or has spurious/inconsistent hits**

Specifically:

(a) From the description in Methods under "Sampling, Ciliate isolation, culturing, and cell-sorting", it is not clear whether all the cells that were ultimately sequenced originated from the same clone (i.e. the same well in the 96-well plate described in line 389). Could the authors confirm whether this was the case?

**Yes. All the sorted cells originated from the same ciliate clone. A single-cell was isolated and cleaned (without removing all the environmental bacteria). The ciliate single-cell divided and we established a mono-clonal ciliate culture that we used for the cell sorting and sequencing. This culture grew but only for a relatively short period. We could not establish a long term culture.**

(b) What % of genes have in-frame coding UAA, UAG, or both? How per gene on average? Counts are given for the conserved genes/domains identified by PhyloFisher or Codetta (lines 192-207), and overall frequencies per codon are addressed later in lines 263 onward, but how often do they occur together in the same genes?

My reasoning behind this is that if genes with both in-frame coding UAA and UAGs are common then it is very unlikely to be the result of chimeric sequence artefacts from whole-genome amplification.

**We have updated the text to include this information. From the PhyloFisher analysis, we had reported that 58 genes contained in-frame UAA codons and 46 genes contained in-frame UAG codons. We have now added the text “Amongst the genes identified by PhyloFisher, 27 contained both an in-frame UAA codon and an in-frame UAG codon.”**

**Additionally, from our annotated gene set, we had reported that 98.6% of genes contain at least one UAA codon and 96.4% of genes contain at least one UAG codon. We have now added text to report how many genes contain both codons “The reassigned codons are widely used across genes with 95.9% of genes containing both a UAA codon and a UAG codon”.**

**The example gene (tubulin gamma chain protein) shown in Figure 1 contains both in-frame UAA codons and in-frame UAG codons, with the UAA codons aligning to lysine and the UAG codons to glutamic acid.**

(c) What is the sequence identity of conserved marker sequences between the individual amplified replicate libraries?

I would naively expect that individual replicates may not have the full set of markers because of uneven amplification, but if the sequences originate from the same clone they should have overlapping coverage of the conserved markers, and these should be +/- identical between replicates (save for allele variants). If so this would support the claim that contaminant sequences were mostly removed during sequence QC and that the cells were clonal.

**We generated an individual assembly for each of the 10 gDNA libraries and calculated average nucleotide identity at the whole assembly level. On average, the 10 assemblies are 99.43% identical to each other, with the least similar pair being 99.37% identical to each other. This level of variation includes not only allelic variants but also sequencing/assembly errors as the individual libraries are relatively low coverage. In terms of assembly alignment coverage (i.e. the fraction of each assembly that is aligned to another assembly), the average value is 76.5% and the value for the lowest pair is 59.1%. We have now also made the individual 10 assemblies available in the Zenodo repository (10.5281/zenodo.7944379) and updated the methods section.**

**Furthermore, as an additional quality control step, we predicted the genetic code for each of the 10 individual genome assemblies and obtained the same predictions that UAA encodes lysine and UAG encodes glutamic acid for all 10 individual assemblies. We also predicted the genetic code for each individual RNA-Seq sample based on individual transcriptome assemblies which yielded consistent predictions.**

(d) Line 392: "Non-axenic" presumably refers to environmental prokaryotes. This also appears to contradict the statement that the cells were "free of any other contaminant" (line 387). Could authors confirm whether they mean "non-axenic but monoeukaryotic"?

**In line 387, when we say "free of any other contaminant” we mean that we isolated a ciliate single-cell from the environmental sample, and the picked ciliate cell was washed 3 times until it was free of any other eukaryotes, but still containing environmental bacteria. In line 392, when we say non-axenic, we mean that the mono-clonal ciliate culture contained environmental bacteria and was monoeukaryotic.**

**We have modified the text in the methods section to say “free from any other eukaryote” and “non-axenic but monoeukaryotic”.**

(e) Lines 448-451: More details should be given on the criteria used to identify and bin out contaminants. MetaBAT typically bins prokaryotic genomes quite well, but not eukaryotic ones. What did the bins look like and how were the eukaryotic ones chosen?

**We routinely use MetaBAT2 to assist with separating bacterial contigs from protist genomes. From our experience we find that it generally performs well but requires careful manual curation. We only use tetranucleotide frequencies when binning single-cell assemblies and not coverage variance as this is heavily skewed due to amplification bias from single-cell amplification. We integrated the binning results from MetaBAT2 with taxonomic classification from tools such as CAT, Blobtools and Tiara, and manually curated the assembly.**

**We have modified both the results and methods section to clarify that the assembly was manually curated to remove contaminant contigs.**

**For example, using CAT, which taxonomically classifies contigs based on blast/diamond hits to open reading frames:**

**The final curated assembly is 69.7 Mb in length.**

**59.5 Mb (85.4%) is classified as Ciliophora.**

**9.7 Mb (13.9%) is unclassified.**

**The remaining 0.5 Mb (0.7%) have inconsistent, low-identity hits to 22 different Eukaryotic and Bacterial phyla (due to lack of closely related species in public databases).**

**Furthermore, we recovered only a single ciliate 18S rRNA gene and the final curated assembly has a unimodal GC content peak with a low BUSCO duplication score and high cDNA mapping rate.**

Minor comments

Line 52: Not strictly true, some germline-limited segments contain mobile elements with coding sequences, e.g. TBE elements in Oxytricha (doi:10.1371/journal.pgen.1003659)

**Thank you for pointing this out. We have rephrased “excision of non-coding sequences” to “excision of micronucleus-limited sequences” to describe the process of macronuclear development more generally.**

Lines 229-231, Supplementary Table 1: Presenting the identity matrix as a distance tree may make it easier to see the pattern of similarity between the tRNAs

**We have added a phylogenetic network of tRNA genes as a supplementary figure to better visualise the relationships between tRNA genes.**

Lines 274-275: Suggest stating the criterion for classifying genes as "highly expressed" on the first mention of this in the Results, although it's explained later on in the Methods.

**We have clarified this in the results section by adding the text: ‘We defined a subset of genes as “highly expressed” based on the 10% of genes with the highest transcripts per million (TPM) values for comparison below.’**

Lines 298-299: What is the frequency of tandem UGA stops in the 3'-UTR in genes with coding-UAA/UAG vs. genes without, and is there a significant difference? The argument in this paragraph is that UAA+UAG reassignment increases selective pressure to minimize translational readthrough. Therefore I think that it would make sense to compare the frequency in genes with and without these codons.

**Following the reviewer’s suggestion, we have looked at tandem UGA stop codons in the 3’-UTR of genes that don’t use UAA and genes that don’t use UAG. We found similar enrichment for in-frame UGA codons at the beginning of the 3’-UTR in these small subsets of genes.**

**To clarify, the hypothesis from the literature is that there may be stronger selective pressure to maintain tandem stop codons in ciliates with reassigned genetic codes, particularly those that use only UGA as a stop codon. Within a genome, we wouldn’t expect a difference if a gene contains UAA/UAG codons.**

Lines 353-354, Figure 5: Suggest marking the internal nodes where genetic code changes likely occurred. At the moment only the leaves of the tree are annotated with the genetic codes of the respective species. This would make it clearer how one counts the numbers of independent origins as reported in the text (e.g. "... a fourth independent origin of UGA being translated as tryptophan").

**We have decided not to label the internal nodes on the phylogeny. We think that deeper sampling will reveal that some of these genetic code changes occurred independently, so we don’t want the figure to be misleading. Also, for the species with the genetic code UAA=Q, UAG=Q and UGA=W, we can’t determine the order of events.**

Lines 371-372: Question out of curiosity (not necessary to address for the manuscript at hand): Do the authors think the recoding of UAA and UAG happened simultaneously in both codons or stepwise, or is there insufficient information to speculate?

**An initial guess would be that it happened as a stepwise process but without deeper sampling of this lineage it is not possible to determine the order of events.**

**This highlights the need for deeper sampling and sequencing across undersampled lineages of ciliates and demonstrates the utility of single-cell OMICs approaches for species that are not yet amenable to culturing.**

Line 395: "10uL" should use the actual symbol for "micro" prefix. Also, the choice of spacing or no spacing between numerical figure and units should be made consistent in manuscript.

**Fixed**

Line 403: "Biotynilated" should be "Biotinylated"

**Fixed**

Line 414 and elsewhere: "2" in MgCl2 should be subscripted

**Fixed**

Lines 419-420: Clarify whether the "r" and "+" symbols are to be read as prefixes or suffixes, i.e. is the modified base the preceding or succeeding one.

**We have clarified in the text that these symbols are to be read as prefixes.**

Table 1: What is the difference between the two sets of BUSCO completeness scores reported? One is given under "Genome assembly" and the other under "Genome annotation", but the annotation is based on the same assembly, right? I'm assuming this has to do with different modes in which BUSCO can be run, but this should be explained in the Methods (lines 452-453, 496-497) and briefly explained in the Table caption.

**Yes this is because we ran BUSCO in two different modes. BUSCO is run in genome mode on the genome assembly and in protein mode on the genome annotation. In genome mode gene prediction is performed by Augustus guided by amino acid BUSCO group block-profiles while in protein mode the gene set described in our methods is the input to BUSCO classification. The superior BUSCO results for the protein mode reflect the superiority of our final annotation over that generated by BUSCO Augustus. We have added text to the methods section and to the table caption to clarify which mode was used.**

**Referee Cross-commenting**

I generally agree with the other reviewers' comments. Specifically I like reviewer #3's suggestion #3 to have a more detailed summary of the codon frequencies, perhaps as a graphic, and to compare the tandem stop frequencies with other ciliate species, especially those with all three canonical stops.

Reviewer #1 (Significance (Required)):

Any new genetic code variant discovered is a cause for celebration! This is a basic biological fact with inherent significance and should be generally interesting to biologists because the rarity of variant codes stands in contrast to the diversity of most biological systems.

This variant code would also stimulate new discussions in the field of genetic code evolution specifically because, as the authors point out, when both UAA and UAG are recoded they both usually encode same amino acid, but here they are recoded to different ones. This is an apparent exception to the "wobble" hypothesis for why these codons often evolve in concert, which was well explained with relevant citations in the Introduction.

For context: My expertise is in genomics and environmental microbiology.

**Reviewer #2 (Evidence, reproducibility and clarity (Required)):**

This study reports the reassignment of the UAA and UAG stop codons to lysine and glutamic acid, respectively, in the ciliate Oligohymenophorea sp PL0344. The paper is nicely written, easy to read and the experimental approach, ideas and questions are easy to follow. The work is technically solid both at the NGS - in house library preparation, sequencing and data interpretation - as well as phylogeny levels. The conclusions are consistent with the comparative genomic and transcriptomic data obtained by the study.

Reviewer #2 (Significance (Required)):

The work extends current knowledge on codon reassignment in ciliates, confirming previous discoveries of existence of very high stop codon assignment flexibility in these organisms. The assignment of UAA and UAG to two different amino acids by two different tRNAs is very interesting and reinforces the idea that stop codon reassignment in ciliates is rather common. It also raises important questions about the parallel evolution of the release factor-1 (eRF1), Lysine and Glutamine tRNAs, as the reassignment requires loss of recognition of both UAA and UAG by eRF1 with parallel appearance of the new Lysine and Glutamic Acid suppressor tRNAs.

The main issue of this work is the inability to cultivate the ciliate Oligohymenophorea sp PL0344 in the laboratory to prepare protein extracts for direct analysis of the amino acids inserted at UAA and UAG sites by Mass Spectrometry. The comparative genomic and transcriptomic data, as well as the identification of cognate tRNA anticodons for UAA and UAG, are likely correct, but provide indirect evidence for the assignment of UAA to Lysine and UAG to Glutamic Acid. This issue is relevant because one cannot exclude the possibility of insertion of other amino acids at UAA and UAG sites beyond Lysine and Glutamic acid, respectively; nor can one exclude the possibility that such amino acids are inserted at high level. The authors do acknowledge the limitations of the unavailability of protein extracts for direct MS analysis of the reassignment, but should consider, in particular in the discussion, the possibility of multiple amino acid insertions in a context where Lysine and Glutamine Acid are the major but not the only amino acid species being inserted at those sites.

Based on my expertise of studying codon reassignments in fungi of the CTG clade, I believe this work is very interesting and appealing to the genetic code community, and is of relevance to the evolution and protein synthesis research communities at large.

**We thank the reviewer for their positive review. They raise an important point about the possibility of amino acids other than lysine and glutamic acid being inserted for UAA/UAG codons which we hadn’t considered. We have added text and relevant references to our discussion to highlight this possibility:**

**“Additionally, while the genomic and transcriptomic data provide strong evidence that lysine and glutamic acid are the major translation products of UAA and UAG codons, respectively, we cannot rule out the possibility that other amino acids are (mis)incorporated at these sites which could be detected using mass-spectrometry [38, 39].”**

**38. Krassowski T, Coughlan AY, Shen X-X, Zhou X, Kominek J, Opulente DA, et al. Evolutionary instability of CUG-Leu in the genetic code of budding yeasts. Nat Commun. 2018;9:1887.**

**39. Mordret E, Dahan O, Asraf O, Rak R, Yehonadav A, Barnabas GD, et al. Systematic Detection of Amino Acid Substitutions in Proteomes Reveals Mechanistic Basis of Ribosome Errors and Selection for Translation Fidelity. Molecular Cell. 2019;75:427-441.e5.**

**Reviewer #3 (Evidence, reproducibility and clarity (Required)):**

Summary: from genome and transcriptome sequencing of what appears to be a novel ciliate from the class Oligohymenophorea, McGowan et al provide convincing evidence of a protist in which the stop codons UAA and UAG have almost certainly been recoded to specify incorporation of different amino acids (UAA = K; UAG = E) during translation. Several ciliates from different classes use a non-standard genetic code (as do a narrow variety of other protists), but this is an unusual observation in that stop codons which differ only in the wobble position code for different amino acids in the ciliate identified here.

I say 'almost certainly' the stop codons have been recoded in Oligohymenophorea sp. PL0344 because in the absence of being able to retain the ciliate in culture the authors have not been able to complete the proteomics which would unequivocally (a) show stop codons now code for amino acids and (b) confirm the identity of the amino acids now encoded (the authors discuss this issue on p12).

Comments: overall this manuscript is straightforward to read and the analyses realistically taken as far as is realistic in the absence of a continuous culture method. My suggested revisions should be straightforward for the authors to address.

1) The manuscript appears to report the identification and genome/transcriptome sequencing of a novel ciliate species - clarity should be provided by the authors. However, it disappointed me that this manuscript was crafted entirely from nucleotide sequencing. I would have welcomed seeing the morphology of the ciliate identified here and would have anticipated that there was sufficient material to perform microscopy at the light level (for DIC images) and by scanning or transmission electron microscopy.

**Yes, based on the 18S rRNA sequence and phylogenies of protein-coding genes, this is a novel species that hasn’t been described before. The most similar hits to the 18S rRNA gene are to other unnamed/environmental sequences. We haven’t attempted to name or describe this species as we weren’t able to establish a culture, so have referred to it as Oligohymenophorea sp. PL0344. We have clarified in the text that this is a novel, unnamed ciliate species.**

**The genomic and transcriptomic data was generated from a single cell isolate propagated into micro-cultures of 10’s of cells. These were done in the strictest conditions in an attempt to minimise contamination. Consistent with this approach it was not possible to obtain useful SEM/TEM as it would be very hard to recover EM imaging from 10’s of cells (a process that would have drastically reduced our ability to do replete genome sampling). Similarly, our approach to culturing limited our ability to acquire useful DIC images. After discovering that this ciliate uses a novel genetic code, we attempted on a number of occasions to re-isolate the same species from the same and surrounding water bodies but failed.**

2) It is unfortunate that the ciliate could not be maintained in culture (or cryopreserved). Coordinates for the University Parks pond are provided, but I got the impression that this ciliate could be repeatedly isolated. Thus, in the absence of culture methods could the authors indicate the points in the year when the ciliate could be isolated (i.e. is there a season element to when PL0344 could be isolated) and how frequently when sampling was performed could PL0344 be seen? From the environmental sequence data that is publicly available is there any evidence for the presence of PL0344 anywhere else in the world? I'd be surprised if this was a UK-specific ciliate.

**The water sample from which this ciliate was isolated was collected in April 2021. After having sequenced its genome and identifying the genetic code change, we made several attempts to reisolate it from the same pond but were unsuccessful. Regarding the geographic distribution of this ciliate, in the text we mention that the most similar 18S rRNA sequence in GenBank is to an unnamed species recovered in a metabarcoding study in France with 99.81% identity. We assume that this is the same species. We also examined other publicly available environmental datasets such as the PR2/metaPR2 database. The most similar match in the metaPR2 database was to a sequence “OLIGO4_XX_sp”. In the metaPR2 database this sequence is unique to Lake Garda in Italy (sample name: “Lake_Garda-LTER-euphotic-water”). However, this hit was only 98% identical with a partial alignment so we did not discuss it in the text. We agree that it is very unlikely that this is a UK-specific ciliate but cannot determine its geographic range based on the publicly available environmental sequence data, other than the single hit to a sequence from France. We think it is important to stress that it was not the aim of our paper to describe the taxonomy and biogeographical range of this ciliate but rather to report the exciting shift in codon usage.**

3) I felt the statistics presented on pages 13-14 (lines 277-301) for codon usage were a little superficial. It would be helpful to see how frequently other E and K codons are used in PL0344 and ideally to see how similar codon usage differs in the more model ciliates Paramecium, Tetrahymena or Stentor. To complete an analysis and justify/confirm conclusions drawn, I would also like to see how frequently in-frame, downstream stop codons are seen in ciliates where stop codons have NOT been reassigned - although the data in Fig 5 indicates genome/transcriptome sequences are not necessarily complete for many ciliate species (where stop codons are not reassigned), there is certainly more varied data to look at than when Fleming and Cavalcanti published their PLoS One work (which is cited in the manuscript).

**We have shortened this section about UAA and UAG usage, with supplementary table 3 showing usage of all codons in all genes compared to our subset of highly expressed genes.**

**We have also added a sentence stating how many genes contain both in-frame UAA and UAG codons based on the point from Reviewer 1: “The reassigned codons are widely used across genes with 95.9% of genes containing both a UAA codon and a UAG codon.“**

**According to our knowledge, there are no new genome assemblies available for ciliates that use the canonical genetic code since the Fleming and Cavalcanti publication from 2019, certainly not any with annotated gene sets available for comparison. The species in Fig 5 which use the canonical genetic code are all from transcriptome data (other than *Stentor*) that have generally low completeness. We do not think comparison with low-quality transcriptome assemblies would make a fair comparison as they would be biased towards transcripts with higher expression. Furthermore, they likely include many fragmented transcripts which are not suitable for detailed comparisons of the stop codon/3-UTR** **region.**

4) Given the presence of just one stop codon in PL0344 have the authors looked genome-wide at nucleotide composition 5' and 3' to UGA. The nucleotide sequences 5' and 3' to a stop can influence whether read through is and thus potentially limits the frequency of or tendency for unwanted readthrough?

**We thank the reviewer for this suggestion which is something we did not investigate initially but have now added a short section in the manuscript to address. Many studies in model organisms have demonstrated that UGA is the least robust stop codon and the most prone to read through. As the reviewer alludes to, this is particularly interesting for ciliates with reassigned genetic codes that use only UGA as a stop codon. Experimental data from model organisms have shown that the sequence composition surrounding a stop codon can influence the frequency of read through, with the nucleotide immediately downstream of the stop codon (“+4 position”) being particularly important.**

**We have now looked at the sequence composition around stop codons for Oligohymenophorea sp. PL0344 and our results show that cytosine tends to be avoided following the UGA stop codon. From the literature, presence of a cytosine following UGA (i.e., UGAC) leads to a substantial increase in translational read through. Furthermore, when examining the subset of highly expressed genes, there are significantly fewer cases of UGAC when compared to all genes. This trend has previously been reported in *Paramecium* and *Tetrahymena* based on EST data (Salim, Ring and Cavalcanti; 2008).**

**We have added a short section to the text reporting this and a supplementary figure showing a sequence frequency logo around the stop codon for all genes and for the subset of highly expressed genes. We are very cautious, however, that there is a paucity of experimental studies investigating stop codon robustness in ciliates. While several publications hypothesise that read through may happen at higher rates in ciliates due to a combination of factors (e.g., ERF-1 mutations, presence of tandem stop codons, competition from suppressor/near-cognate tRNA genes, etc..) we are careful not to speculate without experimental evidence.**

Reviewer #3 (Significance (Required)):

Strengths - I found this a straightforward manuscript to read - aside from the interesting and unexpected observation about genetic code use in PL0344, Fig 5 draws together a lot of earlier published information into an easily accessible form - I felt this a particularly useful part of the manuscript.

I don't feel the absence of proteomics to back up the genome/transcriptome analysis is a notable limitation - it's perhaps frustrating but it's not a limitation. However, the work does perhaps inevitably feel a little bit observational - there's not really a lot of insight or new insight into why the genetic code can be revised in some microbial eukaryotes - in contrast, for instance, to a recently published study of the aptly named Blastocrithidia nonstop. McGowan et al's manuscript, however, will be of interest and should be formally published.

Descriptions of organisms that have tweaked the standard genetic code are not new; coupled to the limited insight into why the genetic code can be rewritten so readily in ciliates, this limits the general appeal of the work. However, the study executed is rigorous and it should be of interest to a wide variety of protistologists, evolutionary cell biologists, and researchers in the translation field.
