# Supplementary material for: Identification of a non-canonical ciliate nuclear genetic code where UAA and UAG code for different amino acids
Source: PLoS Genet. 2023 Oct 5;19(10):e1010913. doi: 10.1371/journal.pgen.1010913 (PMC10553269; doi:10.1371/journal.pgen.1010913)
Supplement: Attachment — Submitted filename: Response Letter - Review Commons resubmission.docx [file pgen.1010913.s010.docx]

**Revision of the research article entitled 'Identification of a Non-Canonical Ciliate Nuclear Genetic Code Where UAA and UAG Code for Different Amino Acids'**

**Corresponding author(s):**

Dr David Swarbreck,

Professor Neil Hall,

Professor Thomas Richards

Requested minor revisions and our response is provided below.

**Thank you for considering our manuscript for publication in PLOS Genetics. We have incorporated your three suggested changes.**

*1. Figure 5: I agree with Reviewer 1’s request that you should label the internal branches in Figure 5 where genetic code changes probably occurred, and I didn’t find your response to this point satisfactory. The statement (line 350) that you “mapped the genetic code reassignments onto the phylogeny” implies that you identified the branches where the changes happened, so please mark them. You say that you don’t want the figure to be misleading but if you don’t mark the branches where you infer that genetic code changes occurred, the justification for statements such as “the translation of UAR as glutamine has evolved at least 5 times” (line 354) is somewhat unclear and open to misinterpretation by readers. In cases where you can’t determine the order of events, you can show them as happening simultaneously.*

**We have modified Figure 5 as suggested and labelled internal branches to indicate when a genetic code change was inferred to have occurred. We have also edited the figure caption to reflect this.**

*2. Your response to Reviewer 3 about the source of the ciliate contains some information that I think should be added to the manuscript’s Methods section (line 379), specifically that it was isolated in April 2021 and that you were unable to find it again.*

**We have added this additional information to the methods section:**

**“Surface water was collected from a margin of an artificial freshwater pond at Oxford University Parks (51°45'51.0"N 1°15'04.5"W), Oxford (UK) in April 2021…”**

**and**

**“…We attempted to establish a long-term culture of Oligohymenophorea sp. PL0344 but were unsuccessful. The culture grew but only for a relatively short period. We also attempted to re-isolate the same ciliate species from the same and surrounding water bodies but failed. We relied on a single-cell/low-input based approach to generate genomic and transcriptomic data.”**

*3. Abstract line 26, please add the word “uncultured”, i.e. change to “a novel uncultured ciliate”.*

**Added**.
